# Wavelet-Decoupled Spatiotemporal Network for Stock Return Prediction

**DOI:** 10.3390/e28050548

**Published:** 2026-05-12

**Authors:** Lei Liao, Chao Wang, Jun Wang, Yinchao Liao, Yanjie Lai

**Affiliations:** 1 School of Finance, Southwestern University of Finance and Economics, Chengdu 611130, China; 122020204056@smail.swufe.edu.cn (L.L.); yl1123@smail.swufe.edu.cn (Y.L.); 2 School of Computing and Artificial Intelligence, Southwestern University of Finance and Economics, Chengdu 611130, China; charlewang@vip.163.com; 3 School of Management Science and Engineering, Southwestern University of Finance and Economics, Chengdu 611130, China; 4 College of Economics and Management, Beijing University of Technology, Beijing 100124, China; lyj13693377646@emails.bjut.edu.cn

**Keywords:** stock prediction, information decomposition, spatiotemporal encoder, cross attention

## Abstract

Stock price prediction is a challenging problem in quantitative investment, as financial markets generate complex, noisy, and dynamic time series containing heterogeneous signals. Short-term fluctuations usually exhibit greater uncertainty and stronger local variation, whereas long-term trends convey relatively stable and persistent information shaped by market and macroeconomic conditions. However, most existing methods struggle to distinguish these two components effectively, often leading to interference between short-term fluctuations and longer-term trends. In addition, they fail to capture dynamic temporal dependencies and cross-stock information propagation while preserving the causal structure of financial time series. To tackle these issues, we propose the Wavelet-Decoupled Spatiotemporal Network (WaveDSTN). It leverages wavelet transformation to decompose stock returns into high-frequency and low-frequency information, corresponding to short-term fluctuations and long-term trends, respectively. This decomposition enables the model to learn complementary predictive patterns more effectively. Furthermore, WaveDSTN incorporates a Dual-Path Spatiotemporal Encoder to capture complex temporal dependencies and evolving cross-stock information propagation while preserving temporal order and causal consistency. Extensive experiments demonstrate that WaveDSTN achieves significant improvements over existing methods, showing that explicitly modeling trend and fluctuation components can enhance predictive accuracy and reduce uncertainty in stock return forecasting.

## 1. Introduction

Stock returns prediction is a fundamental of quantitative investment, with applications ranging from portfolio optimization to algorithmic trading. Nevertheless, accurate forecasting remains difficult because financial markets are highly dynamic and noisy, and stock returns are shaped by multiple interacting sources of information, including macroeconomic indicators, firm-level disclosures, geopolitical events, and investor sentiment [1,2]. The core challenge of stock return prediction lies in extracting useful predictive information from mixed market signals while suppressing irrelevant disturbances. In practice, such complexity is largely reflected in two complementary components of market behavior: long-term trends and short-term fluctuations [3]. Long-term trends aggregate relatively stable information associated with broader market conditions and macroeconomic forces, and therefore evolve more smoothly over extended horizons [4]. In contrast, short-term fluctuations are often driven by localized shocks, rapid information arrivals, trading activity, and sentiment shifts, which introduce stronger uncertainty and richer local variation. Since these two components carry different types of predictive information, effective stock return prediction depends on the ability to distinguish, preserve, and jointly model them [5].

Traditional methods, including decision trees and support vector machines, have been widely employed to predict stock returns [6,7,8,9]. While these approaches leverage statistical relationships, they heavily rely on manually engineered features and often struggle to capture the nonlinear and dynamic relationships inherent in stock market data. Moreover, they cannot effectively distinguish between long-term trends and short-term fluctuations.

With the advent of deep learning, researchers have made significant progress in stock price prediction by leveraging the powerful representation capabilities of neural networks [10,11]. Two predominant directions have emerged: (1) Time correlation. Stock price movements are caused by continuous changes in supply and demand, and time trend changes have obvious dependencies. Representative models include recurrent and convolutional neural networks, which learn stock-price dynamics from individual time-series features. [12,13,14]. (2) Stock correlation. Different stocks in the market form complex and dynamic dependencies due to various factors, such as division of labor and industry status, and stock-price fluctuations affect one another [15,16]. The fusion of stock spatiotemporal features through graphs or self-attention mechanisms can effectively enhance prediction capabilities [2,17]. However, these methods face two limitations.

First, existing studies often fail to adequately exploit the distinct predictive information contained in trend and fluctuation components. Trend information reflects relatively stable long-horizon movement and captures the overall direction of market evolution, whereas fluctuation information corresponds to short-term deviations around the trend and contains rich local variation associated with transient market changes. When these two components are directly mixed or only crudely decomposed, useful predictive patterns may be weakened by information interference, causing complementary signals to be diluted or obscured [15,18,19]. As a result, previous methods often cannot fully utilize the complementary information contained in trend and fluctuation components.

In financial markets, information does not remain isolated within individual stocks, but can propagate across stocks over time through shared fundamentals, market sentiment, industry linkages, and common external shocks [20,21]. Cross-stock relationships are therefore time-varying rather than fixed, and features at different time steps encode distinct temporal states. Moreover, information exchange among stock representations in a forecasting setting must respect causal order: earlier information can influence later states, but not the reverse. Nevertheless, many correlation-mining pipelines either enforce time alignment across series [22], which compresses stock-specific temporal heterogeneity, or treat dependencies as static and omit step-specific representations [23], thereby obscuring causal structure and weakening the model’s ability to capture time-varying information propagation across stocks.

To address these challenges, we propose the Wavelet-Decoupled Spatiotemporal Network (WaveDSTN), a framework for stock return prediction that separately models long-term trends and short-term fluctuations. Specifically, WaveDSTN employs a wavelet transform to decompose stock returns into high-frequency and low-frequency components, corresponding to fluctuation and trend information, respectively. The use of wavelet decomposition is motivated by the representation learning difficulty caused by mixed-frequency return signals. The original return series contains both relatively persistent low-frequency movements and high-frequency variations. If the raw return sequence is directly encoded as a single input, the model has to learn heterogeneous information with different temporal patterns and noise levels within the same representation space. This may weaken the extraction of stable predictive information and increase the interference between trend-related and fluctuation-related signals. Wavelet decomposition provides a multi-resolution representation before neural encoding, allowing the model to learn trend-related and fluctuation-related representations more explicitly. Therefore, in WaveDSTN, wavelet decomposition is not treated merely as a preprocessing step, but as a representation learning strategy for structuring heterogeneous market information. On this basis, WaveDSTN further incorporates a Dual-Path Spatiotemporal Encoder, which combines the temporal representation capability of RWKV with the relational modeling strength of attention mechanisms. This design not only captures evolving dependencies among stocks but also facilitates the modeling of information propagation across stocks and over time while preserving temporal order under causal constraints. Our main contributions are summarized as follows:

(1) We propose a wavelet-decoupled stock return prediction framework that explicitly separates stock returns into trend and fluctuation components. Unlike existing methods that usually model the original return series as a single mixed signal, the proposed framework decomposes heterogeneous market information into low-frequency and high-frequency components. This design helps reduce the interference between relatively stable long-term information and volatile short-term variations, thereby providing cleaner and more informative inputs for return prediction.
(2) We introduce a Dual-Path Spatiotemporal Encoder to jointly model temporal information evolution and cross-stock information propagation. The temporal path adopts RWKV-based causal sequence modeling to preserve the chronological order of financial time series and avoid future information leakage. The spatial path uses an adaptive attention-based relation learning mechanism to capture dynamic cross-stock dependencies. Compared with static graph-based models or purely temporal models, this design allows WaveDSTN to better capture time-varying market relationships under causal constraints.
(3) We design a cross-attention mechanism to model the interaction between trend and fluctuation components. Instead of treating the two decomposed components as independent signals, the proposed mechanism allows the model to adaptively learn how long-term trends and short-term fluctuations influence each other under different market conditions. This improves the model’s ability to utilize complementary information from different frequency components.
(4) Through extensive experiments, we demonstrate the superiority of WaveDSTN over state-of-the-art methods, achieving substantial improvements in both ranking metrics and portfolio-based indicators. The results show that separating and jointly modeling trend and fluctuation information can improve predictive performance and enhance the utilization of complementary market information.

## 2. Related Work

Stock price prediction is a central problem in finance due to its practical importance and complexity. Existing approaches can be broadly categorized into traditional machine learning methods and deep learning-based techniques. This section reviews the key developments in these areas, highlighting their strengths, limitations, and relevance to our proposed framework.

### 2.1. Machine Learning Methods

Traditional machine learning methods—such as decision trees, support vector machines (SVMs), and ensemble approaches like random forests and gradient boosting machines—have been used in stock return prediction for their interpretability, ease of implementation, and computational efficiency [6,7,8,24]. These models typically transform historical market and fundamental data into manually engineered features, including technical indicators (e.g., moving averages, RSI) and fundamental ratios (e.g., P/E, debt-to-equity). While ensemble learners, such as XGBoost, have improved non-linear fitting by combining multiple weak learners, and SVMs can capture non-linear decision boundaries via kernel methods [25], the representation capacity of such models is still constrained by the scope and quality of handcrafted inputs. Unsupervised learning methods, such as k-means clustering and principal component analysis (PCA), have also been employed to preprocess financial data by grouping similar stocks or reducing high-dimensional feature sets [26,27]. Additionally, Bayesian models offer a probabilistic framework for capturing dependencies and dynamically updating predictions based on new data [28,29]. Despite these advancements, traditional methods often struggle to adapt to the complex and dynamic nature of financial markets due to their reliance on manually engineered features, limited ability to capture temporal dependencies, and scalability issues when dealing with large datasets or rapidly changing market conditions. These limitations underscore the need for more advanced techniques that can better model the intricate, spatiotemporal interdependencies inherent in stock markets.

### 2.2. Deep Learning for Stock Prediction

With the rise of deep learning, researchers have explored the representation capabilities of neural networks to model complex behaviors in stock prices. Existing methods primarily focus on two aspects: temporal correlation modeling and stock correlation modeling. Temporal correlation modeling aims to capture sequential dependencies in stock price movements. Recurrent neural networks (RNNs) and their variants, such as Long Short-Term Memory (LSTM) networks and Gated Recurrent Units (GRUs), have been widely used to process time-series data [12,13]. These models excel at capturing temporal patterns but often suffer from vanishing gradients when dealing with long-term dependencies. Convolutional neural networks (CNNs) have also been applied to stock prediction, leveraging their ability to detect local patterns in time-series data [14]. However, CNNs are inherently limited in modeling long-range temporal dependencies. Stock correlation modeling focuses on understanding the interactions and dependencies among stocks. Graph-based approaches and self-attention mechanisms have emerged as powerful tools in this domain. Graph neural networks (GNNs) represent stocks as nodes and their relationships as edges, enabling the modeling of inter-stock dependencies [2,17]. Self-attention mechanisms, such as those employed in the Transformer architecture, further enhance modeling capabilities by capturing both local and global dependencies [23]. Despite their success, these methods often fail to incorporate dynamic temporal features and causal relationships across time steps, limiting their ability to fully capture the complex interplay of temporal and spatial features in stock data.

While these methods have advanced the field, they face several critical limitations. Traditional methods are constrained by their reliance on manual feature engineering and their inability to model complex spatiotemporal interactions. Deep learning-based approaches, though more effective, often treat stock price movements as a whole, failing to distinguish between long-term trends and short-term fluctuations. This oversight leads to mutual interference between the two components, thereby reducing the prediction accuracy. Furthermore, many existing methods oversimplify the temporal dependencies among stocks, neglecting the causal relationships inherent in financial time series.

## 3. Preliminaries

Based on existing works on stock market analysis [30], we adopt a commonly used framework, panel data analysis. Under this setting, historical returns, along with 158 factor-based features of multiple stocks, are utilized to forecast their future returns. Formally, let $x_{t}^{i}\in R^{158}$ denote the 158-dimensional feature vector of stock i at time step t, where $i\in \left\{1,\ldots ,N\right\},$ and N is the number of stocks. Stacking all stocks features at time step t gives $X_{t}=\left\{x_{t}^{1},..,x_{t}^{2},\ldots x_{t}^{N}\right\}\in R^{N\times 158}$ Given a historical dataset: $X=\{X_{1},\ldots ,X_{T_{1}}\}\in R^{N\times T_{1}\times 158}$ of length $T_{1}$, the objective is to predict the next-period cross-sectional returns ${\hat{Y}}_{T_{1}+1}\in R^{N}$.

## 4. Methodology

### 4.1. Overall Architecture

**Figure 1:**
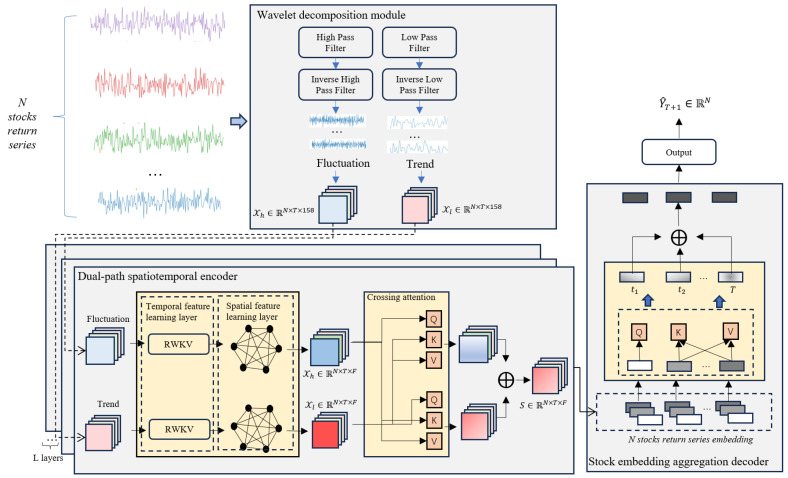
The overview of the WaveDSTN framework, which primarily consists of three parts: the Wavelet decomposition module, the Dual-path spatiotemporal encoder, and the Stock embedding aggregation decoder.

As illustrated in Figure 1, the proposed architecture consists of three key modules: the Wavelet Decomposition Module, the Dual-Path Spatiotemporal Encoder, and the Stock Embedding Aggregation Decoder. The Wavelet Decomposition Module processes historical stock return series using discrete wavelet transforms (DWT) to separate low- and high-frequency components, corresponding to stable long-term trends and volatile short-term fluctuations, respectively. By decomposing the original return series into these two components, the model is able to separate mixed information with different dynamic characteristics and reduce interference between them. The decomposed components are then reconstructed through inverse filtering, concatenated with multifactor features, and projected into high-dimensional representations for downstream modeling. The Dual-Path Spatiotemporal Encoder serves as the core of the framework, comprising a temporal feature learning layer and a spatial feature learning layer. The temporal layer utilizes the RWKV model, which maintains temporal causality through its RNN-like structure while leveraging attention mechanisms for efficiency, allowing the model to capture sequential dependencies within individual stocks. The spatial layer adopts a Graph Attention Network (GAT) with an adaptive adjacency matrix to dynamically model cross-stock dependencies and information propagation in the market. In addition, a cross-attention mechanism is introduced to capture the dynamic exchange between trend and fluctuation information, allowing the model to adaptively emphasize the more informative component under different market conditions. Finally, the Stock Embedding Aggregation Decoder consolidates temporal and spatial representations through a temporal attention layer, aggregating relevant historical information into a unified stock embedding. This embedding is then fed into a linear predictor to generate predictions for stock returns.

### 4.2. Wavelet Decomposition Module

Separating long-term and short-term dynamics requires an appropriate decomposition method. Traditional techniques such as moving averages [31] or the Hodrick–Prescott (HP) filter [32] rely on fixed smoothing rules and often fail to capture local shocks in financial data. In contrast, the Discrete Wavelet Transform (DWT) provides a flexible decomposition framework in both the time and frequency domains, allowing the model to separate mixed information with different dynamic characteristics and to retain informative local patterns more effectively. This property makes wavelets particularly suitable for financial markets, where non-stationarity, volatility clustering, and abrupt regime shifts are common [3,5,33]. Based on this motivation, we employ DWT to disentangle stock returns into trend and fluctuation components, so that relatively stable information and rapidly varying information can be represented separately, thereby reducing interference between them and providing cleaner inputs for subsequent modeling.

To extract the long-term and short-term temporal features from historical stock return data, we apply the Discrete Wavelet Transform (DWT) to decompose the input sequence X into two distinct components: low-frequency and high-frequency parts, which correspond to long-term and short-term market patterns, respectively [34]. The low-frequency part captures smoother, stable trends over longer horizons, while the high-frequency part reflects short-term, volatile fluctuations driven by noise or shocks. The transformation of X is given in Equation (1).

(1) $${\bar{X}}_{l}=gX,{\bar{X}}_{h}=hX,$$

where g and h represent the low-pass and high-pass filters applied in the DWT process, following this, both sub-series ${\bar{X}}_{l}$ and ${\bar{X}}_{h}$ are downsampled to half their original temporal resolution. To recover the original length, we apply the inverse low-pass and high-pass filters $g^{T}$ and $h^{T}$ for upsampling. The resulting sequences are then integrated with the 158-dimensional Alpha158 factor features through concatenation. To prepare these fused features for downstream learning, we project them into a unified latent space using fully connected layers, which produce the transformed representations $X_{l}$ and $X_{h}$. The computation is formalized as:

(2) $$X_{l}=W^{g}g^{T}{\bar{X}}_{l}+b^{g},X_{h}=W^{h}h^{T}{\bar{X}}_{h}+b^{h},$$

where $W^{g}$, $W^{h}$ and $b^{g}$, $b^{h}$ denote the learnable weights and biases associated with the projection layers.

### 4.3. Dual-Path Spatiotemporal Encoder

#### 4.3.1. Temporal Feature Learning Layer

We model temporal correlations for each stock by capturing interactions across time steps t in its feature sequence $X_{i}$. Unlike prior work [23] that uses self-attention over the entire lookback window, we adopt the RNN-based attention architecture RWKV [35] as an efficient alternative to the Transformer [36]. RWKV offers three advantages for financial time series. First, its recurrent formulation preserves temporal order and avoids the need for positional encodings, allowing sequential information to evolve naturally over time. Second, its causal attention [37] restricts step t to attend only to the current and past steps, which prevents future information leakage and ensures that information flow remains consistent with market causality. Third, its time-recursive score update with a time-dependent softmax achieves linear complexity in time and memory, enabling efficient modeling of temporal information over long horizons. In our framework, the same RWKV block is used for both the trend and fluctuation paths to maintain a consistent dual-path information modeling scheme.

**Figure 2:**
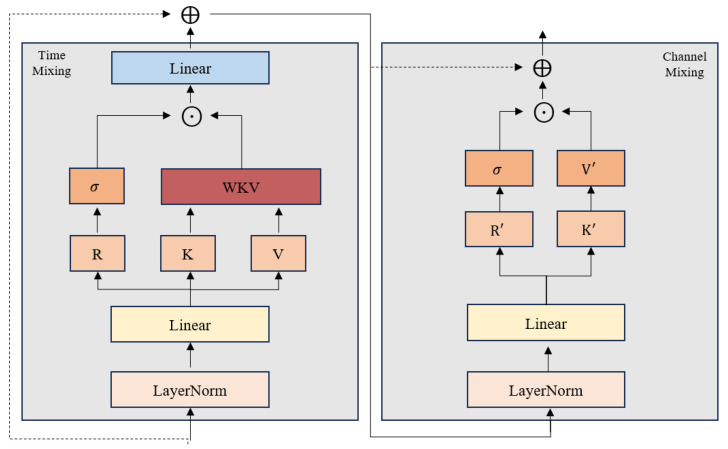
The RWKV structure.

As shown in Figure 2, the RWKV architecture comprises two main parts, namely a time-mixing block and a channel-mixing block. Each sub-block jointly processes features from the current step and its preceding steps, employing a token-shift mechanism to integrate temporal context in a concise and interpretable manner.

In the time-mixing block, the input at time step t is combined with the previous step using the learnable parameters $\mu_{r}$, $\mu_{k}$, $\mu_{v}$. These combined inputs are then linearly mapped to obtain the receptance vector r, the keyword vector k, and the value vector v:

(3) $$r_{t}=W_{r}\cdot \left(\mu_{r}⊙X_{i,t}+\left(1-\mu_{r}\right)⊙X_{i,t-1}\right),$$

(4) $$k_{t}=W_{k}\cdot \left(\mu_{k}⊙X_{i,t}+\left(1-\mu_{k}\right)⊙X_{i,t-1}\right),$$

(5) $$v_{t}=W_{v}\cdot \left(\mu_{v}⊙X_{i,t}+\left(1-\mu_{v}\right)⊙X_{i,t-1}\right).$$

In RWKV, the WKV operator plays a role analogous to self-attention while achieving linear time and space complexity. It incorporates a time-dependent mechanism for updating attention scores, which can be expressed as:

(6) $$wkv_{t}=\frac{\sum_{i=1}^{t-1}e^{-\left(t-1-i\right)w+k_{i}}⊙v_{i}+e^{u+k_{t}}⊙v_{t}}{\sum_{i=1}^{t-1}e^{-\left(t-1-i\right)w+k_{i}}+e^{u+k_{t}}}.$$

Here, w and u are trainable parameters, t∈[1,T] is the time stamp index, ⊙ denotes element-wise product. The vector u introduces a preference at first occurrences, encouraging higher weights when a token is seen for the first time. w is a per-head, channel-wise time-decay parameter that controls how attention diminishes over time across channels. A multi-head variant is used as $wkv_{t}=concat\{{wkv}_{t}^{1},...,{wkv}_{t}^{N}\}$, where N is the number of heads. The output vector $o_{t}$ at time t obtained by the WKV operator is defined as:

(7) $$o_{t}=W_{o}\cdot \left(\sigma \left(r_{t}\right)⊙wkv_{t}\right),$$

where σ denotes the squared ReLU activation function [38].

The channel-mixing block performs a nonlinear combination across feature channels, similar to the time-mixing block. The variables $k^{\prime }$ and $r^{\prime }$ follow the computation in Equation (4). The output $o_{t}^{\prime }$ differs from the time-mixing case and is formulated as:

(8) $$o_{t}^{\prime }=\sigma \left(r_{t}^{\prime }\right)⊙\left(W_{v}^{\prime }\cdot Max{\left(k_{t}^{\prime },0\right)}^{2}\right),$$

Finally, we obtain the local embedding $h_{i,t}$,t∈[1,T] that retains information at step t and aggregates dependencies across different time steps, enabling the model to learn temporal correlations for stock i.

#### 4.3.2. Spatial Feature Learning Layer

In financial markets, stock movements are not isolated but are connected through a complex network of dependencies. From an information transmission perspective, shocks, sentiment changes, and market signals arising in one stock can propagate to related stocks through shared economic fundamentals, sectoral linkages, and investor behavior. One prominent manifestation of this process is momentum spillover, in which price movements or volatility in one stock transmit to others and may further amplify their responses. To accurately model such dynamics, we incorporate a Graph Attention Network (GAT) equipped with an adaptive adjacency matrix. This design captures the evolving relationships among stocks and explicitly integrates momentum spillover effects into the modeling process [39].

To capture the dynamic nature of momentum spillover, we design an adaptive adjacency matrix. Unlike static adjacency matrices that rely on predefined relationships (e.g., industry classification or fundamental features), the adaptive adjacency matrix $A_{t}$ dynamically evolves based on the feature representations of stocks. At each time step t, the relation strength is computed as:

(9) $$R_{i,j}^{t}=LeakyReLU\left(a_{r}^{⊤}W_{r}\left[h_{i}^{t},h_{j}^{t}\right]\right),$$

where $h_{i}^{t}$, $h_{j}^{t}$ are the firm embeddings after extracting the temporal features. We concatenate $h_{i}^{t}$ and $h_{j}^{t}$ and project the result to $R^{F^{\prime }}$ using a linear map $W_{r}\in R^{F^{\prime }\times 2F}$. The projected vector is then transformed to a scalar score with a vector $a_{r}\in R^{F^{\prime }}$, which measures the connection strength between the two firms. To make scores $R_{i,j}^{t}$ comparable across all firms, we apply a Softmax normalization:

(10) $${\overset{\sim }{A}}_{i,j}^{t}={Softmax}_{j}\left(R_{i,j}^{t}\right)=\frac{\exp\left(R_{i,j}^{t}\right)}{\sum_{k\in N,k\neq i}exp\left(R_{i,k}^{t}\right)},$$

where ${\overset{\sim }{A}}_{i,j}^{t}$ is the normalized connection weight from stock j to i at time t. It automatically adjusts to changes in market conditions, such as shifts in sector correlations or the emergence of new market trends. This adaptability is critical in financial markets, where static assumptions about stock relationships may lead to outdated or inaccurate predictions.

After we have obtained the inferred adjacency matrix, we can use the GAT model to model the spatial relationships between firms. In a traditional graph attention network, the momentum spillover from firm j to firm i can be calculated as:

(11) $$s_{i}^{t}=\sigma \left(\sum_{j,j\neq i}^{N}\;\underset{\mathrm{spillovers}\;\mathrm{from}\;j\;\mathrm{to}\;i}{\underbrace{A_{i,j}W_{s}h_{j}^{t}}}\right),$$

where $W_{s}\in R^{F^{\prime }\times F}$ is a shared weight matrix that linearly maps neighbor attributes to higher-level features. $A_{i,j}$ denotes the normalized relation between firms i and j, The function σ(·) is the sigmoid. We replace the original relation matrix A with the learned adaptive matrix $\tilde{A}$.

(12) $$s_{i}^{t}=\sigma \left(\sum_{j,j\neq i}^{N}\;\underset{\mathrm{spillovers}\;\mathrm{from}\;j\;\mathrm{to}\;i}{\underbrace{{\overset{\sim }{A}}_{i,j}^{t}W_{s}h_{j}^{t}}}\right).$$

To capture the heterogeneity of stock relationships and spillover dynamics, we employ multi-head attention, where multiple attention heads compute independent adjacency matrices and aggregate features from diverse perspectives. The combined representation for stock i is:

(13) $$s_{i}^{t}={\|}_{m=1}^{M}\sigma \left(\sum_{j,j\neq i}^{N}\;{\tilde{A}}_{i,j}^{t,\left[m\right]}W_{s}^{\left[m\right]}h_{j}^{t}\right).$$

Finally, we obtain a representation $S_{l}{\in R}^{N\times T_{1}\times F}$ about the trend term and a representation $S_{h}{\in R}^{N\times T_{1}\times F}$ about the volatility term, respectively. These representations encapsulate the long-term trends $S_{l}$ and short-term fluctuations $S_{h}$, respectively. However, in financial markets, trends and fluctuations are not independent; they interact dynamically, and their relative dominance varies over time. To capture these interactions and determine which component plays a dominant role, we design a Cross-Attention Mechanism.

#### 4.3.3. Cross-Attention Layer

The cross-attention mechanism enables us to model the mutual influence between $S_{l}$ and $S_{h}$ by dynamically estimating how these two components affect each other. Specifically, we treat one component as the query and the other as the key and value pair, enabling a bidirectional interaction. For the trend component $S_{l}$ influenced by the volatility component $S_{h}$, the updated representation ${\tilde{S}}_{l}$ is computed as:

(14) $${\tilde{S}}_{l}=Softmax\left(\frac{Q_{l}\cdot K_{h}^{⊤}}{\sqrt{d}}\right)\cdot V_{h},$$

where $Q_{l}=W_{l}^{q}S_{l}$, $K_{h}=W_{h}^{k}S_{h}$, $V_{h}=W_{h}^{v}S_{h}$.

Similarly, the updated representation ${\tilde{S}}_{h}$ for the volatility component influenced by the trend component is:

(15) $${\tilde{S}}_{h}=Softmax\left(\frac{Q_{h}\cdot K_{l}^{⊤}}{\sqrt{d}}\right)\cdot V_{l},$$

where $Q_{h}$, $K_{l}$, $V_{l}$ are defined analogously.

Finally, we obtain a representation ${\tilde{S}}_{l}$ about the trend term and a representation ${\tilde{S}}_{h}$ about the volatility term, respectively. We add them up to get a spatial–temporal representation of the stock $Z{\in R}^{N\times T_{1}\times F}$.

(16) $$Z={\tilde{S}}_{l}+{\tilde{S}}_{h}.$$

### 4.4. Stock Embedding Aggregation Decoder

In the stock embedding aggregation decoder, a temporal attention layer pools each stock’s temporal features to produce the final embedding $\tilde{Z}{\in R}^{N\times F}$ [19]. This step focuses on and consolidates the most informative temporal signals. For stock i with embedding $z_{i,t}$, the last-day embedding $z_{i,T}$ serves as the query over all time steps in the window. The attention weights and the aggregated representation are computed as:

(17) $$\lambda_{u,t}=\frac{\exp\left(z_{i,t}^{T}W_{\lambda }z_{i,T}\right)}{\sum_{t=1}^{T}\;exp\left(z_{i,t}^{T}W_{\lambda }z_{i,T}\right)},$$

(18) $$e_{u}=\sum_{t\in \left[1,T\right]}\;\lambda_{i,t}z_{i,t}.$$

Finally, the stock-level embedding $e_{u}$ is passed to the predictor g(·) to generate the forecast. We use a linear head for g and train with mean-squared error (MSE).

(19) $${\hat{r}}_{u}=g\left(e_{u}\right),\;\mathrm{Loss}=\sum_{i\in S}\;MSE\left(r_{i},{\hat{r}}_{i}\right).$$

## 5. Experiment

### 5.1. Experiment Setup

#### 5.1.1. Dataset

We evaluate our framework using the Chinese and US stock markets, specifically the CSI300 and S&P500 stock datasets. The selection of these datasets is consistent with recent stock prediction literature, which provides a comparable empirical setting with prior studies and allows us to test the cross-market applicability of the proposed framework [23,31,40]. The time range is from 1 January 2010 to 31 December 2020. We utilize the Alpha158 dataset from the Qlib platform [41], which comprises 158 technical features extracted from price–volume data. The lookback window length T is set to 20. We split the data into training (from 1 January 2010 to 31 December 2017), validation (from 1 January 2018 to 31 December 2018), and test (from 1 January 2019 to 31 December 2020) datasets. We summarize the statistics of these datasets in Table 1. To ensure the quality and consistency of the input data for training and evaluation, we performed several preprocessing steps, including standardization and handling of missing values.

#### 5.1.2. Evaluation Metrics

To evaluate the performance of the compared methods, we use the rank information coefficient (RankIC) and RankICIR (Rank Information Coefficient Information Ratio) as metrics, which is the dominant ranking metric in finance. RankIC is the Spearman rank correlation coefficient between the ranking of predicted stock returns and the ranking of realized stock returns. [42,43]. It is computed as the Spearman rank correlation coefficient between the two rankings: $RankIC=Spearman\left(R_{pred},R_{true}\right).$ Therefore, its value ranges from −1 to 1. A RankIC close to 1 indicates that the predicted ranking is highly consistent with the realized ranking, while a RankIC close to −1 indicates an inverse ranking relationship. A higher RankIC indicates better ranking accuracy, but not necessarily lower numerical prediction error. RankICIR is the ratio of the mean RankIC to its standard deviation, providing a measure of the model’s stability and consistency in predictive performance over time. A higher positive RankICIR suggests that the model not only achieves better ranking performance on average but also maintains this performance more consistently across different periods. It can be calculated as: $RankICIR=\frac{mean\left(RankIC\right)}{std\left(RankIC\right)}$.

#### 5.1.3. Baselines

We evaluate WaveDSTN against a set of representative baselines covering tree ensembles, recurrent and convolutional sequence models, and graph-based methods that are widely used for stock prediction. The baselines were introduced in Related Work and are implemented with comparable training setups. In total, we compare with seven models:

- LightGBM [44]: A gradient-boosted decision-tree method noted for speed, efficiency, and suitability for large datasets.
- LSTM [45]: A gated recurrent architecture designed to retain long-range temporal information, widely adopted for forecasting time series and for NLP tasks.
- GRU [13]: The gated recurrent unit, a lighter alternative to LSTM with fewer gates, usually achieves comparable accuracy with lower computational cost.
- ALSTM [18]: An LSTM variant equipped with an attention module so the model can place higher weight on informative time steps.
- TCN [14]: A temporal convolutional network using causal (often dilated) 1-D convolutions to obtain long receptive fields for sequence modeling.
- ADGATs [39]: A homogeneous graph attention approach that integrates attribute-based aggregation to capture momentum spillovers among related firms.
- STGCN [46]: It captures spatial–temporal correlations by combining GCN with causal convolutions.

#### 5.1.4. Parameter Settings

The proposed WaveDSTN model is implemented using the PyTorch version 1.10.1 and optimized with the Adam optimizer over 100 training epochs, with a batch size of 8. In the model architecture, the multi-head attention mechanism consists of 8 heads, and the base feature dimension *d* is set to 256. The spatiotemporal encoder comprises 5 layers. The training process starts with an initial learning rate of 0.0001, which is adjusted using a decay scheduler with a rate of 0.1. To reduce overfitting, dropout regularization is applied with a rate of 0.2. To ensure the robustness of our evaluation, each method included in the experiments was trained five times with different random initializations. The average performance across these runs during the testing phase is reported to minimize fluctuations caused by initialization variance. Each training session took approximately 6 h on a single NVIDIA A100 GPU.

### 5.2. Comparative Analysis of Predictive Performance

Table 2 reports the cross-sectional stock-ranking performance of all compared methods measured by RankIC and RankICIR. Overall, WaveDSTN achieves the best performance on both the CSI300 and S&P500 datasets in terms of RankIC and RankICIR. On the CSI300 dataset, LightGBM achieves a RankIC of 0.012 and a RankICIR of 0.272. Although LightGBM can capture nonlinear feature relationships, its performance is limited because it does not explicitly model temporal dependencies or cross-stock information transmission. Temporal models, including LSTM, GRU, ALSTM, and TCN, achieve higher RankIC values ranging from 0.027 to 0.039, suggesting that modeling historical sequential patterns improves cross-sectional return prediction. However, these models mainly focus on the time-series evolution of individual stocks and do not sufficiently capture dynamic relationships among stocks. Graph-based models perform better, with ADGATs and STGCN achieving RankIC values of 0.046 and 0.042, respectively, which indicates that incorporating cross-stock dependencies and information propagation is useful for return prediction. WaveDSTN further improves upon these baselines, achieving a RankIC of 0.057 and a RankICIR of 0.568 on the CSI300 dataset. Compared with the strongest baseline, ADGATs, WaveDSTN improves RankIC by 0.011 and RankICIR by 0.154, corresponding to relative improvements of 23.91% and 37.19%, respectively. These improvements suggest that WaveDSTN produces predicted returns that are more consistent with realized cross-sectional return rankings and that its predictive signal is more stable across periods. This advantage can be attributed to its decomposition-based spatiotemporal design: instead of modeling stock returns as a single mixed signal, WaveDSTN separates trend and fluctuation components and then models their temporal evolution and cross-stock propagation. A similar pattern is observed on the S&P500 dataset. LightGBM obtains a RankIC of 0.012 and a RankICIR of 0.211, while temporal models achieve RankIC values between 0.017 and 0.029. Graph-based models again show stronger performance, with ADGATs reaching a RankIC of 0.042 and a RankICIR of 0.387. WaveDSTN achieves the highest RankIC and RankICIR, with values of 0.054 and 0.582, respectively. These results show that the advantage of WaveDSTN is consistent across both the Chinese and US markets. Overall, the comparison across model categories indicates that each additional modeling dimension contributes to better performance: temporal models improve upon traditional machine learning by capturing sequential dependence, graph-based models further benefit from cross-stock relational information, and WaveDSTN achieves the best results by jointly modeling multi-scale return information, temporal dependence, and cross-stock interactions. Therefore, the empirical results support the effectiveness of the proposed framework rather than merely showing numerical superiority over individual baselines.

### 5.3. Robustness Check

To further evaluate the robustness of WaveDSTN under more recent market conditions, we conduct an additional out-of-time test using data from 2021 to 2025. This period differs from the original test period because it includes post-COVID-19 market adjustments, inflationary pressure, monetary policy changes, and heightened geopolitical uncertainty. These factors make stock return prediction more challenging and provide a stricter test of model robustness. Table 3 reports the post-2020 robustness results on the CSI300 and S&P500 datasets. Compared with the original 2019–2020 test period, the overall RankIC and RankICIR values decline for most models. This is consistent with the more challenging market environment after 2020. Although the absolute performance decreases, WaveDSTN still achieves the highest RankIC and RankICIR on both datasets. On the CSI300 dataset, WaveDSTN obtains a RankIC of 0.046 and a RankICIR of 0.471, outperforming the strongest baseline, ADGATs, which achieves a RankIC of 0.037 and a RankICIR of 0.356. On the S&P500 dataset, WaveDSTN achieves a RankIC of 0.047 and a RankICIR of 0.483, also higher than ADGATs, which obtains 0.036 and 0.265, respectively. These results suggest that WaveDSTN maintains a relative advantage in cross-sectional ranking performance during the post-2020 period, although the prediction task becomes more difficult under more volatile market conditions. Therefore, the robustness test supports the generalizability of the proposed framework, while also showing that market regime changes can reduce the absolute level of predictive performance.

### 5.4. Ablation Study

To investigate the effects of different components within the WaveDSTN model, this study compares it against five distinct variants. Each variant removes or modifies a specific component to analyze its contribution to the overall performance. The ablation experiments were conducted on the CSI300 and S&P500 datasets using RankIC and RankICIR as evaluation metrics. The five variants are described as follows:

- “w/o” decomposition: The wavelet decomposition module is removed, and the raw stock return series is used as input. Without separating stock returns into trend (low-frequency) and fluctuation (high-frequency) components, the model processes all features as a unified series.
- “w/o” trend: This variant excludes the trend component (low-frequency) from the input. Only the fluctuation component (high-frequency) is processed by the model.
- “w/o” fluctuation: In contrast to the second variant, this version removes the fluctuation component (high-frequency) from the input, retaining only the trend component (low-frequency).
- “w/o” cross-attention: This variant removes the cross-attention mechanism, preventing the model from capturing the interaction between trend and fluctuation components. The trend and fluctuation are modeled independently without considering their mutual influence.
- “w/o” aggregation: The final temporal aggregation step, which consolidates temporal features into a single embedding for each stock, is removed. Instead, the feature of the last time step is directly used for prediction.

As shown in Table 4, the ablation results provide more direct evidence for the contribution of each component in WaveDSTN. Removing the wavelet decomposition module leads to a clear decline in performance. On the CSI300 dataset, RankIC decreases from 0.057 to 0.041, and RankICIR decreases from 0.568 to 0.415. On the S&P500 dataset, RankIC decreases from 0.054 to 0.038, and RankICIR decreases from 0.582 to 0.382. This result indicates that separating stock returns into trend and fluctuation components helps the model learn more useful return representations than directly modeling the original mixed return series. Removing the trend component causes the largest performance decline on both datasets, with RankIC decreasing to 0.024 on CSI300 and 0.022 on S&P500. This suggests that low-frequency trend information plays an important role in the prediction task. Removing the fluctuation component also reduces performance, with RankIC decreasing to 0.025 on both datasets, indicating that high-frequency fluctuation information provides complementary predictive signals. The removal of the cross-attention mechanism also weakens model performance, with RankIC decreasing from 0.057 to 0.049 on CSI300 and from 0.054 to 0.044 on S&P500. This suggests that modeling the interaction between trend and fluctuation components is useful, rather than treating the two components as fully independent inputs. Finally, removing the temporal aggregation module leads to a smaller but consistent decline, indicating that aggregating historical temporal representations contributes to the final stock embedding. Overall, the ablation results show that the performance of WaveDSTN is not driven by a single component alone. Instead, the results support the combined design of wavelet-based decomposition, trend–fluctuation interaction, and temporal aggregation for stock return prediction.

### 5.5. Hyperparameter Sensitivity Analysis

To assess the impact of hyperparameter choices on the performance of the WaveDSTN model, we conducted a sensitivity analysis focusing on four critical hyperparameters within the Dual-Path Spatiotemporal Encoder and overall architecture.

**Figure 3:**
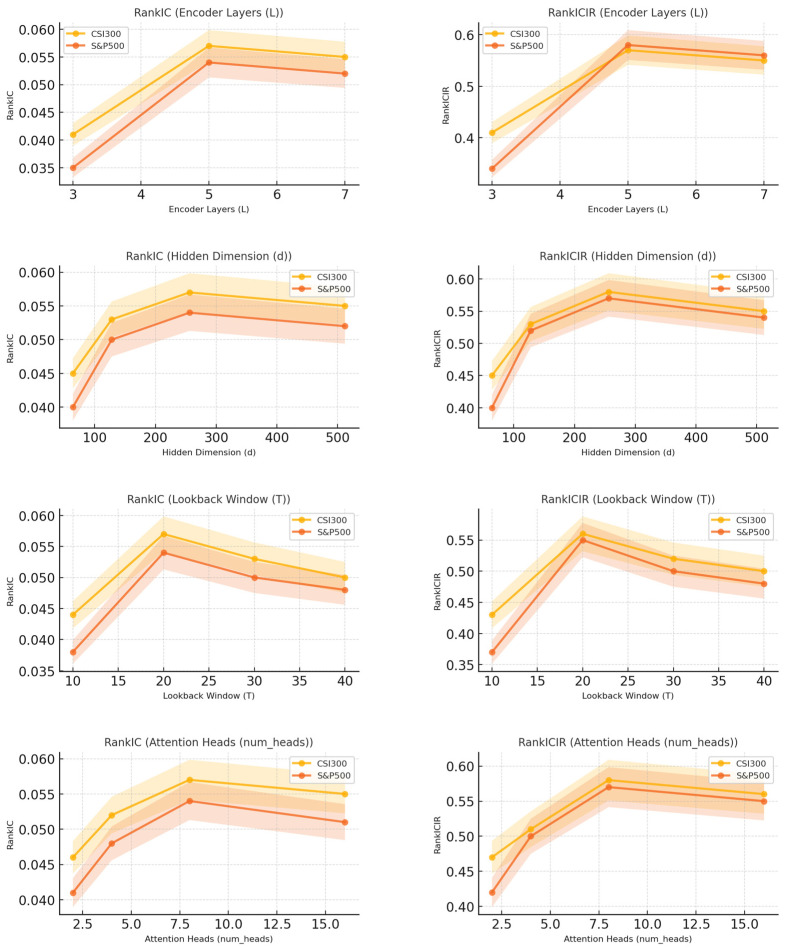
The results of hyperparameter sensitivity.

The hyperparameter sensitivity analysis examines the effects of four key hyperparameters on the performance of WaveDSTN: the number of encoder layers (L), hidden dimension size (d), lookback window size (T), and the number of attention heads (num_heads). In the experiments, one hyperparameter is varied at a time while the others are kept at their default values. Specifically, L is tested over {3, 4, 5, 6, 7}, d over {64, 128, 256, 512}, T over {10, 20, 30, 40}, and num_heads over {2, 4, 8, 16}. As shown in Figure 3, the model is sensitive to these hyperparameter choices. For the number of encoder layers, the best performance is obtained at L = 5, while further increasing the depth does not lead to additional improvement and may introduce redundant model complexity. For the hidden dimension size, d = 256 achieves the best performance. Smaller hidden dimensions may limit the representation capacity of the model, whereas larger dimensions increase computational cost without clear performance gains. For the lookback window size, T = 20 provides the best result, suggesting that this setting captures sufficient historical information while avoiding excessive noise from a longer input window. For the number of attention heads, num_heads = 8 performs best, indicating a suitable balance between relational representation capacity and model complexity. Overall, the sensitivity analysis shows that WaveDSTN performs best under a moderate model size and input window. Excessively small settings may lead to insufficient representation capacity, while overly large settings may introduce unnecessary complexity and noise.

### 5.6. Investment Simulation

To assess the real-world applicability of WaveDSTN, we construct portfolios based on the predictions of different models and compare their performance through backtesting. We adopt the TopK-Drop strategy to maintain a portfolio on each trading day. Specifically, on trading day t, TopK-Drop constructs an equal-weight portfolio $P_{t}$ consisting of k stocks selected according to the ranking of predicted returns, subject to the turnover constraint $|P_{t}\cap P_{t-1}|\;\geq \;k-n$. Here, k denotes the number of stocks held in the portfolio, while n controls the maximum number of stocks that can be replaced between two consecutive trading days. In our experiment, we set k=50 and n=5, meaning that at least 45 stocks are retained from the previous portfolio and at most 5 stocks are replaced at each rebalancing period.

**Figure 4:**
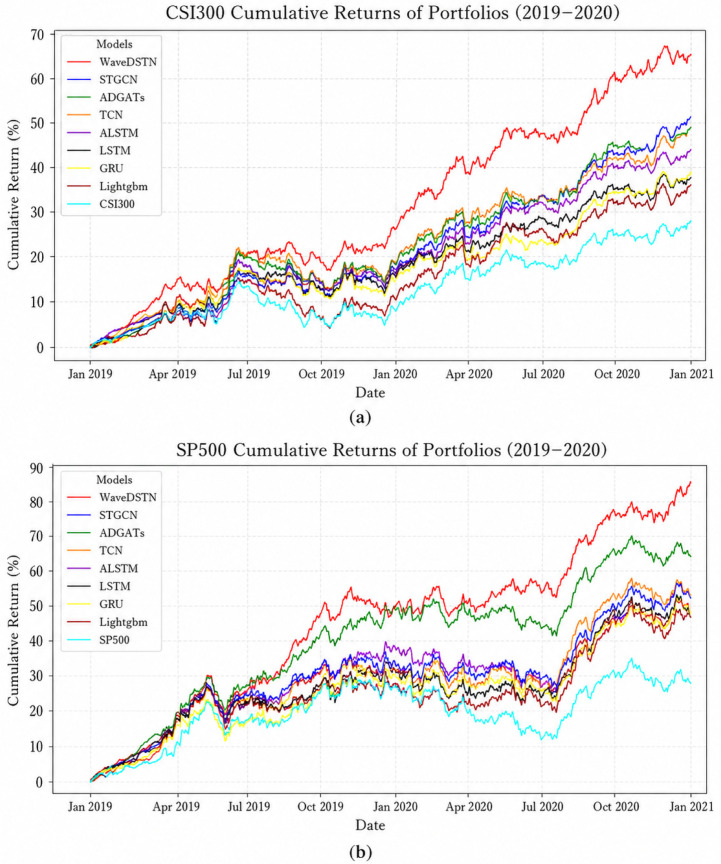
Cumulative return comparison. (**a**) Cumulative return comparison in CSI300; (**b**) Cumulative return comparison in S&P500.

As shown in Figure 4a,b, the portfolios constructed based on WaveDSTN predictions achieve the highest cumulative returns on both the CSI300 and S&P500 datasets. On the CSI300 dataset, WaveDSTN reaches a cumulative return of more than 60% by the end of 2020, clearly outperforming the baseline models. Graph-based methods such as STGCN and ADGATs achieve relatively stronger performance than traditional sequence models, but their cumulative returns remain lower than those of WaveDSTN. A similar pattern is observed on the S&P500 dataset, where WaveDSTN achieves a cumulative return of more than 80%, while the strongest baseline models remain at a lower level. After adding the CSI300 and S&P500 index benchmarks, the results also show that the portfolio performance of WaveDSTN is not solely driven by the overall market trend, but is related to the predictive signals generated by the model.

Table 5 further reports the annualized return (AR) and Sharpe ratio (SR) of different portfolio strategies. On the CSI300 dataset, WaveDSTN achieves the highest AR of 28% and SR of 2.3, compared with 26% and 2.1 for the strongest baseline, ADGATs. On the S&P500 dataset, WaveDSTN also performs best, with an AR of 35% and an SR of 3.1, while ADGATs achieve an AR of 27% and an SR of 2.4. These results suggest that WaveDSTN not only improves cumulative portfolio returns, but also provides better risk-adjusted performance under the adopted TopK-Drop backtesting setting.

Overall, the investment simulation indicates that the stronger ranking performance of WaveDSTN can be translated into portfolio-level gains. However, these results should be interpreted within the limits of the experimental setting. The backtest does not fully account for transaction costs, liquidity constraints, market impact, and other implementation frictions. Therefore, the results demonstrate the potential practical value of WaveDSTN for portfolio construction, rather than guaranteeing the same level of performance in real trading environments.

## 6. Discussion

In this section, we further discuss the effectiveness and limitations of WaveDSTN. The main idea behind WaveDSTN is that stock returns should not be treated as a single homogeneous signal. From a representation learning perspective, directly feeding the original return series into a prediction model may force the model to learn different types of market information within a single mixed representation. However, trend and fluctuation components have different frequency characteristics and noise levels. Low-frequency trend information is usually smoother and more persistent, while high-frequency fluctuation information is more irregular and sensitive to short-term shocks. When these components are not separated, useful low-frequency signals may be disturbed by high-frequency variations, and informative short-term patterns may also be weakened in the learning process. Wavelet decomposition provides a multi-resolution representation of financial time series by separating the original return sequence into low-frequency and high-frequency components. This is useful for predictive learning because it allows the model to construct more specialized representations for different types of market information: the low-frequency component can support the learning of trend-related representations, while the high-frequency component can support the learning of fluctuation-related representations. In this sense, wavelet decomposition is not only a preprocessing step, but also a representation learning strategy that reduces mixed-frequency interference and provides more structured inputs for subsequent spatiotemporal modeling.

Regarding the model’s limitations, this study has several limitations that should be acknowledged. First, although our model leverages wavelet decomposition to separate market information, the current framework relies on predefined decomposition scales to construct the trend and fluctuation components. Financial markets contain information at different time scales. Future work could extend the current trend–fluctuation decomposition to a richer multi-scale structure, so that the model can capture short-, medium-, and long-term return patterns more effectively. Second, our current empirical evaluation focuses primarily on predictive accuracy (e.g., MSE, IC) and idealized portfolio returns. In real-world trading environments, factors such as transaction costs, bid-ask spreads, and market slippage could discount the actual profitability of the generated signals. Future studies could integrate reinforcement learning algorithms to directly optimize portfolio construction while explicitly accounting for realistic trading frictions. Third, WaveDSTN relies exclusively on structured market data (i.e., historical price and volume). It does not incorporate unstructured alternative data, such as financial news, corporate filings, or social media sentiment, which are known to be significant drivers of short-term market fluctuations. Future work could extend the framework into a multi-modal architecture by fusing these unstructured textual signals to further enhance predictive performance.

## 7. Conclusions

This study aims to improve stock return prediction by addressing two key challenges in financial time-series modeling: the mixed nature of heterogeneous market information and the dynamic propagation of information across stocks. To this end, we propose WaveDSTN, a Wavelet-Decoupled Spatiotemporal Network that decomposes stock returns into trend and fluctuation components and models their temporal evolution, cross-stock propagation, and interaction within a unified framework. The empirical results on the CSI300 and S&P500 datasets show that WaveDSTN achieves better performance than traditional machine learning models, temporal sequence models, and graph-based spatiotemporal baselines in terms of RankIC and RankICIR. The results indicate that WaveDSTN generates predicted return signals with stronger cross-sectional information content and greater temporal stability. The ablation study further confirms the contribution of the main components of the framework. In particular, wavelet decomposition helps separate heterogeneous market information, while the spatiotemporal encoder and cross-attention mechanism improve the modeling of temporal dynamics, cross-stock relationships, and trend–fluctuation interactions. The investment simulation also shows that portfolios constructed based on WaveDSTN predictions achieve stronger cumulative return and risk-adjusted performance under the adopted TopK-Drop strategy.

The significance of this study is twofold. From a methodological perspective, the results show that combining frequency-domain information decomposition with spatiotemporal representation learning can improve stock return prediction. From a practical perspective, the findings suggest that predicted return signals generated by WaveDSTN can be useful for portfolio construction and quantitative investment analysis.

Despite its strengths, WaveDSTN has several limitations. It is currently evaluated on two large-cap equity universes and mainly relies on price–volume-based Alpha158 features, which may limit its applicability to broader markets and information sources. The investment simulation also uses a simplified backtesting setting without fully considering transaction costs, liquidity constraints, short-selling limits, or market impact. Future research could extend the model to broader asset universes, incorporate alternative data, and evaluate its performance under more realistic trading conditions.

## Figures and Tables

**Table 1:** Dataset details.

Market	CSI300	S&P500
Stocks	463	507
Training period	1 January 2010–31 December 2017	
Validation period	1 January 2018–31 December 2018	
Test period	1 January 2019–31 December 2020	
Features	158	

**Table 2:** Cross-sectional returns prediction performance of the compared methods.

Datasets	Model	RankIC	RankICIR
CSI300	LightGBM	0.021 ± 0.0018	0.272 ± 0.018
	LSTM	0.027 ± 0.0021	0.322 ± 0.021
	GRU	0.029 ± 0.0020	0.331 ± 0.020
	ALSTM	0.034 ± 0.0024	0.340 ± 0.024
	TCN	0.039 ± 0.0019	0.334 ± 0.022
	ADGATs	0.046 ± 0.0018	0.414 ± 0.026
	STGCN	0.042 ± 0.0028	0.375 ± 0.024
	WaveDSTN	0.057 ± 0.0023	0.568 ± 0.022
S&P500	LightGBM	0.012 ± 0.0023	0.211 ± 0.017
	LSTM	0.018 ± 0.0021	0.252 ± 0.019
	GRU	0.017 ± 0.0019	0.261 ± 0.018
	ALSTM	0.026 ± 0.0022	0.318 ± 0.021
	TCN	0.029 ± 0.0024	0.306 ± 0.020
	ADGATs	0.042 ± 0.0017	0.387 ± 0.025
	STGCN	0.034 ± 0.0026	0.325 ± 0.022
	WaveDSTN	0.054 ± 0.0022	0.582 ± 0.021

Each model is trained five times with different random seeds. We report the mean and standard deviation of RankIC and RankICIR across these independent runs. Statistical significance is evaluated by paired *t*-tests between WaveDSTN and the strongest baseline on each dataset. The *p*-values for the *t*-tests were all less than the critical confidence value (0.05), indicating that the superior performance of the proposed approach is statistically significant.

**Table 3:** Post-2020 robustness results for cross-sectional stock return prediction.

Datasets	Model	RankIC	RankICIR
CSI300	LightGBM	0.012 ± 0.0016	0.118 ± 0.018
	LSTM	0.019 ± 0.0021	0.214 ± 0.023
	GRU	0.020 ± 0.0020	0.226 ± 0.022
	ALSTM	0.025 ± 0.0025	0.271 ± 0.021
	TCN	0.031 ± 0.0020	0.289 ± 0.024
	ADGATs	0.037 ± 0.0019	0.356 ± 0.019
	STGCN	0.034 ± 0.0028	0.331 ± 0.025
	WaveDSTN	0.046 ± 0.0024	0.471 ± 0.023
S&P500	LightGBM	0.010 ± 0.0023	0.154 ± 0.018
	LSTM	0.015 ± 0.0022	0.205 ± 0.021
	GRU	0.016 ± 0.0020	0.218 ± 0.019
	ALSTM	0.022 ± 0.0018	0.286 ± 0.022
	TCN	0.026 ± 0.0023	0.302 ± 0.020
	ADGATs	0.036 ± 0.0022	0.365 ± 0.024
	STGCN	0.032 ± 0.0027	0.334 ± 0.026
	WaveDSTN	0.047 ± 0.0021	0.483 ± 0.022

Each model is trained five times with different random seeds. We report the mean and standard deviation of RankIC and RankICIR across these independent runs. Statistical significance is evaluated by paired *t*-tests between WaveDSTN and the strongest baseline on each dataset. The *p*-values for the *t*-tests were all less than the critical confidence value (0.05), indicating that the superior performance of the proposed approach is statistically significant.

**Table 4:** Ablation study.

Datasets	Model	RankIC	RankICIR
CSI300	“w/o” decomposition	0.041 ± 0.0024	0.415± 0.021
	“w/o” trend	0.024 ± 0.0025	0.281± 0.023
	“w/o” fluctuation	0.025 ± 0.0023	0.325± 0.022
	“w/o” cross-attention	0.049 ± 0.0024	0.478± 0.024
	“w/o” aggregation	0.052 ± 0.0022	0.512± 0.021
	WaveDSTN	0.057 ± 0.0023	0.568 ± 0.022
S&P500	“w/o” decomposition	0.038 ± 0.0025	0.382± 0.022
	“w/o” trend	0.022 ± 0.0024	0.211± 0.024
	“w/o” fluctuation	0.025 ± 0.0023	0.272± 0.021
	“w/o” cross-attention	0.044 ± 0.0022	0.452± 0.023
	“w/o” aggregation	0.045 ± 0.0023	0.484± 0.022
	WaveDSTN	0.054 ± 0.0022	0.582 ± 0.021

Each model is trained five times with different random seeds. We report the mean and standard deviation of RankIC and RankICIR across these independent runs. Statistical significance is evaluated by paired *t*-tests between WaveDSTN and the strongest baseline on each dataset. The *p*-values for the *t*-tests were all less than the critical confidence value (0.05), indicating that the superior performance of the proposed approach is statistically significant.

**Table 5:** The portfolio performance of AR and SR.

Datasets	Model	AR	SR
CSI300	LightGBM	0.10	0.9
	LSTM	0.14	1.1
	GRU	0.15	1.2
	ALSTM	0.17	1.3
	TCN	0.19	1.6
	ADGATs	0.26	2.1
	STGCN	0.23	1.9
	WaveDSTN	0.28	2.3
S&P500	LightGBM	0.12	0.8
	LSTM	0.15	1.0
	GRU	0.16	1.1
	ALSTM	0.18	1.4
	TCN	0.20	1.5
	ADGATs	0.27	2.0
	STGCN	0.25	1.8
	WaveDSTN	0.35	2.5

## Data Availability

The data supporting this study were obtained from the open-source Qlib platform. Publicly archived datasets (e.g., Alpha158) are available through the Qlib repository and documentation at: https://github.com/microsoft/qlib and https://qlib.readthedocs.io/, accessed on 8 October 2025. In addition, for reproducibility purposes, the data used in this study have been archived on Zenodo and are available at: https://doi.org/10.5281/zenodo.19150237.
